# Content comparison of long-term care instruments based on the international classification of functioning, disability and health (ICF)

**DOI:** 10.1186/s12877-023-03824-2

**Published:** 2023-03-17

**Authors:** Yan Gao, Jingpu Zhao, Xiangxiang Liu, Xiaohua Xie, Yulong Wang

**Affiliations:** 1 Department of Rehabilitation Medicine, Shenzhen Second People’s Hospital, First Affiliated Hospital of Shenzhen University, No. 3002, Sungang West Road Futian District, Shenzhen, China; 2 Department of Nursing, Shenzhen Second People’s Hospital, First Affiliated Hospital of Shenzhen University, Shenzhen, China

**Keywords:** Long-term care, Older adults, International Classification of Functioning, Disability and Health (ICF), Instrument

## Abstract

**Background:**

Ageing poses a huge challenge to the Chinese social welfare system. However, a national long-term care (LTC) instrument has not been established yet. This study aimed to analyse and compare the content of six selected LTC instruments based on the linkage of the International Classification of Functioning, Disability and Health (ICF) to provide a content reference from a functioning perspective for the development of a Chinese national LTC instrument.

**Methods:**

Two trained health professionals performed the linkage analysis according to the refined ICF linking rules. The main concepts included in the items of three international LTC instruments, namely Minimum Data Set 3.0 (MDS 3.0), Initial Assessment Instrument (IAI), and New Assessment Tool for Determining Dependency on Nursing Care (NBA), as well as three Chinese instruments, namely Disability Assessment of Long-Term Care (DA-LTC), Specification for Elderly Care Unified Need Assessment in Shanghai Version 2.0 (SEC-UNA 2.0), and pictorial-based Longshi Scale (LS), were selected and linked to the ICF categories. The six selected LTC instruments were analysed and compared at the levels of ICF components, chapters, and categories.

**Results:**

The main concepts of 340 items of the six LTC instruments were linked to 112 different ICF categories. Within the ICF framework, the ‘*Activities and Participation*’ component was most frequently addressed in the LTC instruments, followed by ‘*Body functions*’, at 52% and 38%, respectively. At the chapter level, ‘*b1 mental functions*’, ‘*d4 mobility*’, and ‘*d5 self-care*’ were addressed by the majority of LTC instruments.

**Conclusion:**

The ICF provides a general reference for the analysis and comparison of different LTC instruments. The findings indicate that all LTC instruments focused on older adults’ physical needs; however, various important issues regarding their psychological and social participation needs were not addressed. Specific for China, the core elements of LTC instruments varied, and the ICF chapters ‘*b1*’, ‘*d4*’, and ‘*d5*’ are recommended to develop a national uniform one.

## Background

Over the past two decades, the ageing population (65 years and older) in China has almost doubled, reaching 144 million (10.5%) in 2015 [1]. It is predicted to sharply increase to 366 million (26.1%) in 2050 [1]. As one of the fastest ageing countries, the rapidly growing and unmet care needs of older persons are a huge challenge to the Chinese social welfare system. Specifically, this indicates a significant requirement for an effective long-term care (LTC) system to ensure appropriate allocation of limited social resources [2].

Based on the experience of developed countries with well-coordinated and cost-effective LTC systems, a key step involves developing the eligibility criteria for LTC benefits and services. Such criteria determine the baseline expenditure needed to run the systems and play a critical role in objectively and fairly deciding the benefits for individuals [3, 4]. Countries that have successfully implemented national LTC services have standard and structured assessment tools—such as the Initial Assessment Instrument (IAI) in Japan [5], New Assessment Tool for Determining Dependency on Nursing Care (NBA) in Germany [4], and Minimum Data Set (MDS) of the International Resident Assessment Instrument-Minimum in the US [6]. However, a nationally uniform assessment tool to determine the eligibility of older persons for LTC services in China has not been established yet [2].

According to the World Health Organization (WHO), LTC proposes to provide service for people who are in need of long-term care to ‘*maintain dignity and an independent daily life routine according to each person’s own level of ability*’ [7]. While developing operational eligibility criteria for LTC, an important issue is identifying the key factors that influence the ageing population to function independently. The methods to develop LTC eligibility criteria generally include expert consultation, literature review, and clinical observation [4, 5]. However, the descriptions of healthy ageing and LTC both point to a biopsychosocial perspective of health, specifically the consideration of functioning and independence in daily activities.

**Fig. 1:**
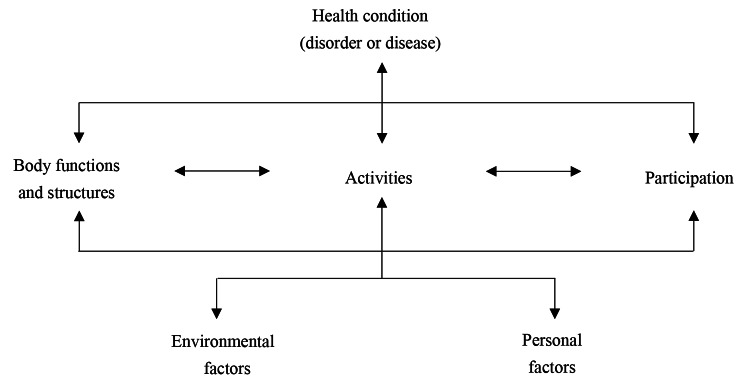
The framework of the ICF

‘Functioning’, as the operational concept of ‘health’ in the International Classification of Functioning, Disability and Health (ICF) framework, is not a stable attribute, but is a fluid and continuous interaction between body functions and structures, activity and participation, as well as contextual factors (Fig. 1) [8]. In the last two decades, functioning and its domains have been widely applied in the collection of national and international health information [9, 10], as well as in policy content evaluation [11]. A major public health goal of the WHO is helping individuals, including the ageing population, attain a high functioning level. Hence, the key factors of LTC from the perspective of functioning are notable.

As an international classification system, the ICF has a systematic infrastructure and universal taxonomy to document people’s functional data. It was conceived as a common language for different disciplines that can be applied in varied settings. Using the proposed transparent linking rules [12, 13], the ICF has been accepted as a reference to compare variation in the collected health information from different perspectives and apply various modes such as technical or clinical examinations, health assessment instruments, and healthcare interventions. The ICF can help health practitioners, researchers, social workers, and policymakers understand diverse health information regarding functioning.

Therefore, this study aimed to analyse and compare LTC instruments’ content based on the linkage of the ICF to provide a reference from a functioning perspective for the development of a Chinese national LTC instrument. The specific aims were (i) to analyse the content of LTC assessments from a functioning perspective by the linkage of the ICF framework and (ii) to compare the similarities and differences between LTC instruments’ content.

## Methods

This study selected six LTC instruments: three international LTC instruments and three newly published or widely applied Chinese instruments. The international instruments were the MDS 3.0 [6], IAI [5], and NBA [4], while the Chinese ones were the Chinese Disability Assessment of Long-Term Care (DA-LTC) [14], Chinese Specification for Elderly Care Unified Need Assessment in Shanghai Version 2.0 (SEC-UNA 2.0) [15], and pictorial-based Longshi Scale (LS) [16].

The established and refined ICF linking rules developed by the ICF Research Branch were used to link the items in the instruments to the most precise ICF categories [12, 13]. Two authors (Gao and Zhao)—both familiar with the concepts, definitions, and structure of the ICF and having linkage experience—performed the linkage separately. Any differences between the two linkers were resolved through discussion. Where agreement could not be reached, the third author made an informed decision. The online ICF Browser (https://apps.who.int/classifications/icfbrowser/) was applied as a resource for linkage [17].

### Linkage of items to the ICF

The ICF has two parts: functioning and disability as well as contextual factors. Functioning and disability refer to the components of body function (*b*), body structure (*s*), and activities and participation (*d*). Contextual factors are divided into the components of the environment (*e*) and an unclassified set of personal factors (*pf*). An example of the ICF cord structure is presented as follows:

**Table:** 

b2	sensory functions and pain	(first- or chapter level)
b280	sensation of pain	(second-level)
b2801	pain in body part	(third-level)
b28010	pain in head and neck	(fourth-level)

To ensure that the linkage process was conducted meaningfully and transparently, the linking process was guided by the ICF linking decision tree [13].

### Instruments

The number of domains/sections, items, published country/region, mode of administration, purpose, and scoring of these instruments are presented in Table 1. The section refers to clusters of items, and the domain refers to themes.

**Table 1:** The Long-term care instruments

Instrument	Country/Region	Administration	Domains/Sections^*^	Number of items	Purpose	Scoring
Minimum Data Set 3.0, MDS 3.0	US-HCFA	Interview/clinical observation	21^*^	152	Planning of care	/
New assessment tool for determining dependency on nursing care, NBA	Germany-ABMH	Interview/clinical observation	8^*^	76	Classified the degrees of dependency	1st degree of dependency (15–29), 2nd degree (30–49), 3rd degree (50–69), 4th degree (70–89), 5th degree of dependency (either 90+, or 90 + and additional specific need constellation).
Initial assessment instrument, IAI	Japan-MHLW	Interview/clinical examination	8	85	Estimated total care minutes per day	Not eligible (≤25minutes), Need support (<30minutes), Level 1(<50minutes), Level 2(<70minutes), Level 3(<90minutes), Level 4(<110minutes), and Level 5(≥110minutes)
Disability assessment of Long-term care, DA-LTC	Chinese-NHSA &MCA	Interview/clinical examination	3	17	Classified the degrees of disability	No impairment, Slightly impairment, Moderate impairment, and Severe impairment I. Severe impairment II, Severe impairment III
Specification for elderly care unified need assessment in Shanghai 2.0, SEC-UNA 2.0	Chinese-SHHSA	Interview/clinical examination	8^*^	83	Classified the degrees of care requirement	Totally dependent, Severe dependent, Moderate dependent, Moderate slight dependent, Slight dependent, Totally independent
Longshi Scale, LS	Chinese-MCA	Interview/clinical observation/ self-administered	1	9	Classified the degrees of self-care	Totally dependent, Severe dependent, Moderate dependent, Moderate slight dependent, Slight dependent, Totally independent

HCFA Health Care Financing Administration; ABMH the Advisory Board and the Ministry of Health; MHLW Ministry of Health, Labor, and Welfare,

NHSA National Healthcare Security Administration; MCA Ministry of Civil Affairs; SHHSA-Shanghai National Healthcare Security Administration

^*^ Sections

The MDS 3.0 is a systematised and standardised multi-dimensional assessment that addresses the problems of LTC facility residents and their potential needs [6]. The data collected from residents can be aggregated to help in planning their care and improving care quality. The MDS 3.0 has 21 sections. This study excludes the identification information and four summary sections (participation in assessment and goal setting, care area assessment summary, correction request, and assessment administration). Therefore, 96 items were included for the linkage.

The IAI has two subscales, physical and mental status, and the use of medical procedures [5]. Trained local government officials completed this form through home visits. Based on standard evaluation scores, the assessors estimated the time needed for the nine care categories (grooming/bathing, eating, toileting, transferring, assistance with instrumental activities of daily living [ADL], behavioural problems, rehabilitation, and medical services) per day, and assigned a care-needs level to applicants.

The NBA has eight modules [4]. Among them, six (mobility, cognition and behaviour, self-care, management of illness-related demands, everyday life, and social contacts) were included in the scoring system. Their scores were weighted and integrated into an overall score between 0 and 100. Five degrees of dependency were identified according to the threshold scores.

The DA-LTC was newly published by the National Healthcare Security Administration and Ministry of Civil Affairs of the People’s Republic of China in 2021 [14]. It has 27 items and is divided into three domains: ADL, cognitive ability, and perceptive and communicative ability. It classifies applicants into six disability levels.

The SEC-UNA version 2.0 has 83 items and is divided into two subscales: applicants’ self-care ability and disease severity [15]. The SEC-UNA classifies applicants into seven degrees (from 0 to 6) depending on the threshold scores of each subscale. This study excluded the items on identification information (items 1–26).

The LS, a pictorial-based self-care assessment tool that has been widely applied in Chinese communities and nursing homes [16], has nine items that evaluate self-care ability in ADLs. It classifies applicants into six degrees of self-care.

### Data analysis

The reliability of the linking process between the two researchers was evaluated using the $\kappa$ statistic and nonparametric bootstrapped 95% confidence intervals. The *κ* values were categorised as follows: $\kappa$ value of 0.00–0.20 = slight agreement, 0.21–0.40 = fair, 0.41–0.60 = moderate, 0.61–0.80 = substantial, and 0.81–1.00 = almost perfect [18].

Content analysis and comparison of selected LTC instruments were based on linked ICF categories. The number of linked ICF categories was calculated and grouped by the ICF components.

## Results

### Linkage process

The main concepts of 340 items of the six LTC instruments were linked to 112 different ICF categories (two *s*-categories, 42 *b*-categories, 58 *d*-categories, five *e*-categories, and five *pf*-categories) based on published linking rules [12, 13]. In the ICF framework, the ‘*d*’ component was most frequently addressed in LTC instruments, followed by ‘*b*’, at 52% and 38%, respectively. A total of 30 concepts, including ‘hallucinations’, ‘delusions’, ‘depression’, and ‘anxiety’, were identified as ‘not defined-mental health (*nd-mh*)’; 48 concepts of disease diagnoses were assigned to ‘*health conditions (nc-hc)*’, and six concepts such as ‘bedfast’, ‘functional rehabilitation potential’, and ‘fall history on admission’ were assigned to ‘*not covered*’.

The results of the $\kappa$ statistic for the agreement between the two researchers were 0.73, and the 95% bootstrapped confidence intervals were 0.52 and 0.91, respectively, presented as substantial reliability.

### Linkage results

Table 2 presents the frequency of items, concepts, and ICF categories for each LTC assessment. It shows that within the ICF framework, the MDS 3.0 comprises all the ICF components. However, none of the other five LTC instruments comprise the ‘*s*’ and ‘*pf*’ components. The MDS 3.0 focused more on the ‘*b*’ component, while the other five LTC instruments more greatly stressed the ‘*d*’ component, with percentages over 50% and even reaching 89.5% in the pictorial-based LS scale.

**Table 2:** Number of items (%) of long-term instruments containing concepts that address categories of a particular ICF component

	MDS 3.0	NBA	IAI	DA-LTC	SEC-UNA 2.0	LS
Number of ICF categories	55	35	32	23	49	19
Number of main concepts	240	76	85	24	80	24
ICF categories per component						
Body structures [n (%)]	2(3.6)	0(0)	0(0)	0(0)	0(0)	0(0)
Body function [n (%)]	26(47.3)	8(22.9)	14(43.8)	9(39.1)	20(40.8)	2(10.5)
Activity and participation [n (%)]	17(30.9)	26(74.3)	17(53.1)	14(60.9)	29(59.2)	17(89.5)
Environment factors [n (%)]	5(9.1)	1(2.9)	1(3.0)	0(0)	0(0)	0(0)
Personal factors [n (%)]	5(9.1)	0(0)	0(0)	0(0)	0(0)	0(0)

MDS 3.0 Minimum Data Set 3.0; NBA New assessment tool for determining dependency on nursing care; IAI Initial assessment instrument; DA-LTC Disability assessment of Long-term care; SEC-UNA 2.0 Specification for elderly care unified need assessment in Shanghai 2.0; LS Longshi Scale

Table 3 presents the selected LTC instruments’ content comparison using the ICF categories as a reference grouped by the components. Within the ICF framework, the ‘*d*’ component was the most frequently addressed in the LTC instruments, followed by the ‘*b*’ component. The ‘*s*’, ‘*e*’, and ‘*pf*’ components scarcely covered 2%, 4%, and 4% of the 112 linked categories, respectively.

**Table 3:** Linkage of the Long-term care instruments to the ICF categories of the components

ICF Category	MDS3.0	IAI	NBA		DA-LTC	SEC-UNA 2.0	LS
Body structure							
s320 Structure of mouth	1						
s810 Structure of areas of skin	6						
Body functions							
b110 Consciousness functions	1						
b1100 State of consciousness	1						
b1140 Orientation to time	1	1	1		1	2	
b1141 Long-term memory	1	2	1			2	
b1142 Orientation to person					1		
b11420 Orientation to self		1	1				
b117 Intellectual functions	1		1			1	
b1266 Psychic stability						1	
b130 Energy and drive functions						1	
b1300 Energy level	1						
b1302 Appetite	1						
b134 Sleep functions	1		1			1	
b1400 Sustaining attention	2						
b144 Memory functions		1	1			3	
b1440 Short-term memory	2	1			1	2	
b1441 Long-term memory	1				1		
b1442 Retrieval of memory	1						
b1470 Psychomotor functions	2						
b152 Emotional functions	2					1	
b1521 Regulation of emotion	2						
b156 Perceptual functions						1	
b1565 Visuospatial perception					1		
b160 Though functions	1					1	
b1641 Organization and planning			1				
b1671 Expression of language			1			1	
b1720 Simple calculation						1	
b210 Seeing functions	1	1			1	1	
b230 Hearing functions performance	1	1			1	1	
b280 Sensation of pain	1						
b3300 Fluency of speech	1						
b4400 Respiration rate	1						
b5105 Swallowing	1	1					
b525 Defecation functions	1					1	1
b5253 Faecal continence	1	1			1	1	
b620 Urination functions							1
b6202 Urinary continence	1	1			1	1	
b710 Mobility of joint functions		1				1	
b7108 Mobility of joint functions, other specified	1						
b7356 Tone of muscles of lower half of body						1	
b760 Control of voluntary movement functions		1					
b7600 Control of simple		1					
b810 Protective functions of the skin		1					
Activity and participation							
d177 Making decision	1		1				
d230 Carry out daily routine		1	1				
d2301 Managing daily routine						1	
d240 Handling stress and other psychological			1				
d310 Communicating with-receiving-spoken messages	1				1		
d315 Communicating with receiving-nonverbal messages	1				1		
d350 Conversation			1				
d3600 Using telecommunication devices						1	1
d398 Communication, other specified		1					
d410 Changing basic body position	1		1			1	1
d4100 Lying down						1	
d4103 Sitting		1				1	
d4104 Standing	1	1	1				
d4106 Shifting the body’s center of gravity		1				1	
d4153 Maintaining a sitting position		1	1			1	
d4154 Maintaining a standing position		2				1	
d420 Transferring oneself	3	1			1		
d450 Walking	1	1					1
d4500 Walking short distances	2				1	1	
d4501 Walking long distances	1					1	
d4508 Walking, other specified	1						
d4551 Climbing			1		1	1	
d4600 Moving around within the home			1				
d4602 Moving around outside the home and other building			1				1
d465 Moving around using equipment	1				1	2	
d470 Using transportation						1	1
d5 Self-care						1	
d510 Washing oneself			1				1
d5100 Washing body parts					1	1	
d5101 Washing whole body	1				1	1	
d520 Caring for body part	1	1	1				1
d5200 Caring for skin						1	
d5201 Caring for teeth					1	1	
d5202 Caring for hair					1	2	
d530 Toileting	1		1		1	1	1
d5300 Regulating urination		1					
d5301 Regulating defecation		1					
d5308 Toileting, other specified					1		1
d540 Dressing	1	1			1		
d5400 Putting on clothes			1			2	
d5401Taking off clothes			1			2	
d550 Eating	1	1	1		1	1	1
d560 Drinking			1				
d570 Looking after one’s health		1	1				
d5708 Looking after one’s health, other specified						1	1
d6200 Shopping			1			1	1
d630 Preparing meals						1	1
d6300 Preparing simple meals			1				
d640 Doing housework			1			1	1
d6402 Cleaning living area		1	1				
d710 Basic interpersonal interactions			1			1	
d720 Complex interpersonal interactions			1				
d860 Basic economic transactions		1	1			1	
d910 Community life	1						
d920 Recreation and leisure			1				
d9205 Socializing							1
d9208 Recreation and leisure, other specified							1
d9209 Recreation and leisure, unspecified							1
Environment							
e1101 Drugs	2						
e120 Products and technology for personal indoor and outdoor mobility and transportation	1						
e1251 Assistive products and technology for communication	2						
e198 Products and technology, other specified	7						
e5800 Health services	31	12	15				
Personal factor							
Importance for daily preferences	8						
Importance for activity preference	8						
Height	1						
Weight	1						
Weight loss	1						

ICF International Classification of Functioning, Disability and Health; MDS Minimum Data Set; IAI Initial Assessment Instrument; NBA New Assessment Tool for Determining Dependency on Nursing Care; DA-LTC Chinese Disability Assessment of Long-Term Care; SEC-UNA Chinese specification for Elderly Care Unified Need Assessment in Shanghai; LS Longshi Scale

In the ICF chapter level, the MDS 3.0 focused on 15 chapters and five other LTC instruments were linked to 7–13 chapters. All six LTC instruments covered the chapters ‘*d4 mobility*’ and ‘*d5 self-care*’. The most frequently addressed chapter was ‘*b1 mental functions*’. Moreover, the six LTC instruments’ distribution showed considerable differences. For example, for the ‘*b*’ component, the MDS 3.0 covered all the chapters except ‘*b8 functions of the skin and related structures*’. However, the NBA only comprised ‘*b1 mental functions*’. For the ‘*d*’ component, the NBA covered all the nine chapters, whereas the MDS 3.0 focused on four chapters—less than four out of the five instruments.

In the ICF category level, only the following three categories and their subcategories (including third-level ICF categories) were covered by all the six selected LTC instruments: ‘*d520 caring for body parts*’, ‘*d530 toileting*’, and ‘*d550 eating*’. Other important categories covered in five of the six LTC instruments were ‘*b110 consciousness functions*’, ‘*b144 memory functions*’, ‘*b525 defecation functions*’, ‘*b620 urination functions*’, ‘*d410 changing basic body position*’, ‘*d510 washing oneself’*, and ‘*d540 dressing*’. Moreover, Table 3 shows the number of times a specific ICF category was identified in the linked LTC instruments. A high number may indicate that the ICF concept was broad or complex; therefore, several concepts of items from an LTC instrument had to be linked to the same ICF category. For example, 31 concepts of the MDS 3.0, 12 concepts of the IAI, and 15 concepts of the NBA were linked to the ICF category ‘*e5800 health services*’.

## Discussion

This study compared and analysed the content of six LTC instruments using the ICF as a reference system. The content comparison shows that ‘*b1 mental functions*’, ‘*d4 mobility*’, and ‘*d5 self-care*’ are at the core of LTC instruments. At the ICF component level, the MDS 3.0 is a comprehensive LTC instrument covering all the components to describe functioning. The three selected Chinese LTC instruments are mainly focused on the ‘*b*’ and ‘*d*’ components, and the contents are varied at the chapter level.

Theoretically, LTC instruments’ content should adhere to the national LTC policies and reflect the conceptual basis to be measured. Based on ICF categories’ linkage, the similarities and differences between the LTC policies could be clearly explained. For example, the NBA policy’s outline focused on a population that showed ‘*dependency on nursing care*’, and the aims of IAI were ‘*to promote seniors’ functional independence, rather than excessive dependency on institutions and the government*’. It could be elucidated that the NBA considered more to compensate for—or manage—applicants’ dependency using personal assistants, while the IAI intended to reduce people’s dependency by improving their functioning. Like this study, most items in the NBA were linked to ICF’s ‘*d*’ component, potentially because most limitations of ‘activities’ and ‘participation’ could be addressed by caregivers. By comparison, nearly half the items in IAI were linked to the ‘*b*’ component because seniors’ physiological or psychological functions could be improved through treatment to reduce dependency.

In China, the Ministry of Human Resources and Social Security published the guidelines of the LTC policy (Trial) in 2016 [19]. The targeted population comprised individuals ‘*who were severely disabled in basic requirements of life and medical care*’. However, unlike some developed countries, such as Japan and Germany, which have clear guidelines to develop national LTC instruments, the key elements of Chinese LTC instruments have not been established yet [2]. In this study, the content of three selected Chinese LTC instruments varied; hence, this may lead to significant differences in the number of eligible applicants. For example, the pictorial-based LS scale did not evaluate applicants’ ‘*mental function*’. However, the estimated number of Chinese older adults ($\ge$60 years) with dementia was 15.3 million in 2019 and is estimated to reach 45.5 million in 2050 [20]. Moreover, the items of SEC-UNA 2.0 and LS Scale were linked to the chapter ‘*d6 domestic life*’, which assessed older people’s abilities of instrumental activity of daily living (IADL), but none were involved in the DA-LTC. According to the data of China Health and Retirement Longitudinal Study (CHARLS) 2018, the percentage of older people with IADL disability was above triple times that of ADL disability, which were 11.1% and 34.8%, respectively [21].

This study revealed that all LTC instruments focused on older adults’ physical needs. Among all the linked ICF categories, more than 80% were about ‘*activities*’, which is conceived as ‘*the execution of specific tasks or actions by an individual*’ and ‘*body functions*’ categories. However, from the biopsychosocial medical model perspective, the psychological and social participation needs of older adults should not be ignored [22–25]. Two large prospective studies conducted in Japan proved that social participation among older adults might decrease the need for LTC and, consequently, reduce future LTC costs [23, 24]. Furthermore, unmet psychological distress among older adults may accelerate the decline of health and functional status and increase the use of emergency health services [25].

This study indicated that some important issues pertaining to older adults were not addressed by most LTC instruments. For example, five out of six LTC instruments did not cover the category ‘*b280 pain*’ even though more than 60% of community-dwelling older adults and up to 80% living in LTC facilities suffer from chronic pain [26, 27]. Chronic pain in older people could lead to impaired ADLs, anxiety, disturbed appetite and weight loss, cognitive disorders, and an increased burden on the healthcare system [28, 29]. Therefore, given the high prevalence of pain and its influence on older adults, it is an undeniably important issue in LTC. Moreover, the three LTC instruments did not cover the category ‘*b134 sleep function*’. Sleep is an essential element for health promotion among older adults. However, sleep disturbances, such as insomnia, are prevalent, ranging from 30 to 48% among the older adult population. Its negative health consequences include decreased quality of life, increased risk of falls, psychological and physical difficulties, and healthcare system costs [30].

As a national LTC instrument, the amount of time required for administration is a critical factor that significantly influences the utility of LTC instruments. This study presented that after linking the items of six selected LTC instruments to ICF categories, the average administration time per ICF category differed greatly. For example, there were 52 and 17 ICF categories linked to the MDS 3.0 and pictorial-based LS scale, respectively. However, the average time to complete the MDS 3.0 and LS scale was around 61.5 and 1.5 min, respectively [6, 31]. Given that the estimated number of older adults (aged > 65 years) in China with disabilities was 18 million in 2020 and is expected to reach 52 million in 2050 [2], the number of persons applying for LTC will increase exponentially. Thus, a time-consuming LTC instrument would reduce its feasibility of use, as it would likely lead to longer waiting times before applications are processed. To help counter a longer waiting period, many health or social service professionals trained in LTC assessment would be required; however, this is not feasible either.

The important limitation in this study is that although applying the ICF linking process can be used to collect different types of health information, all data cannot always be linked to the ICF classification system for comparison. For example, some behavioural objects—such as unsanitary behaviour, collecting items inappropriately, and repeatedly narrating the same story—were not covered in the ICF system.

## Conclusion

To the best of our knowledge, this is the first study to analyse and compare LTC instruments using the ICF as a reference. The linkage results indicate that although the content of LTC instruments varied, all of them focused on older adults’ physical needs. Various important issues regarding older adults’ psychological and social participation needs were not addressed. Specific for China, the content differences among current LTC instruments could lead to a significant difference in the number of eligible applicants. Based on this study’s findings, the core elements of a uniform Chinese national LTC instrument should include the ICF chapters of ‘*b1 mental functions*’, ‘*d4 mobility*’, and ‘*d5 self-care*’.

## Data Availability

The datasets used and/or analyzed during the current study are available from the corresponding author (Yulong Wang) on reasonable request.

## Abbreviations

LTC: Long-term care
IAI: Initial Assessment Instrument
NBA: New Assessment Tool for Determining Dependency on Nursing Care
MDS: Minimum Data Set
DA-LTC: Disability Assessment of Long-Term Care
SEC-UNA: Specification for Elderly Care Unified Need Assessment
LS: Longshi Scale
ADL: Activities of Daily Living
IADL: Instrumental Activities of Daily Living
